# Supplementary material for: Willingness to engage in and current status of social participation among Chinese merchant sailors
Source: PLoS One. 2020 Nov 25;15(11):e0242888. doi: 10.1371/journal.pone.0242888 (PMC7688133; doi:10.1371/journal.pone.0242888)
Supplement: S1 File — (DOCX) [file pone.0242888.s001.docx]

**Data underlying the findings described in the manuscript**

**1 Willingness to participate in public affairs among Chinese merchant sailors, broken down by several groups (%)**

|  |  | Would you like to participate in public affairs in your community? | | | | | | |
| --- | --- | --- | --- | --- | --- | --- | --- | --- |
|  |  | Very willing | Willing | Unwilling | Completely unwilling | Not sure | Total | N |
| Groups | | 14.2 | 53.4 | 20.7 | 1.9 | 9.8 | 100 | 7296 |
| **Age** | less than 30 years old | 17.5 | 53.7 | 19.6 | 2.2 | 7.1 | 100 | 2386 |
|  | 30–39 years old | 12.2 | 53.0 | 23.1 | 2.4 | 9.3 | 100 | 2416 |
|  | 40–49 years old | 13.4 | 53.1 | 20.2 | 1.0 | 12.2 | 100 | 1527 |
|  | 50 years old above | 11.4 | 54.4 | 16.6 | 1.2 | 16.4 | 100 | 625 |
| **Educational background** | Junior high school diploma and below | 10.3 | 45.4 | 19.5 | 3.7 | 21.1 | 100 | 410 |
|  | High school diploma | 13.4 | 53.5 | 19.5 | 1.0 | 12.6 | 100 | 1581 |
|  | Junior college diploma | 15.1 | 52.3 | 22.2 | 2.0 | 8.5 | 100 | 3630 |
|  | Bachelor degree and above | 13.8 | 57.0 | 18.4 | 3.4 | 7.4 | 100 | 1322 |
| **Sailing area** | Ocean-going area | 16.7 | 55.3 | 18.6 | 1.9 | 7.5 | 100 | 4003 |
|  | Coastal area | 10.8 | 50.1 | 23.8 | 2.2 | 13.1 | 100 | 3068 |
| **Types of ships working** | Ordinary cargo ship | 14.9 | 53.7 | 20.4 | 2.0 | 9.0 | 100 | 4801 |
|  | Oil and Gas Ships | 12.5 | 51.8 | 21.6 | 2.3 | 11.8 | 100 | 2104 |
| **Types of contract** | Contract worker | 15.9 | 54.3 | 19.5 | 1.8 | 8.5 | 100 | 4073 |
|  | Dispatched workers | 14.8 | 53.2 | 22.3 | 2.4 | 7.4 | 100 | 1170 |
|  | Individual crew | 9.8 | 50.4 | 22.3 | 2.6 | 14.9 | 100 | 1828 |

Note: Information was unreported for the age of 342 sailors, for the educational background of 353 sailors, for the types of ships working of 391, the sailing area of 225, the working age of 193, and the type of contract for 225.

The same to the following tables.

**2 Concern for national policies across several groups of Chinese merchant sailors (%)**

|  | | Are you concerned about the national policies introduced in recent years? | | | | | |  |
| --- | --- | --- | --- | --- | --- | --- | --- | --- |
|  | | Great concern | Concern | Little concern | No concern | Not sure | Total | N |
| Groups | | 14.1 | 52.2 | 27.8 | 2.2 | 3.7 | 100 | 7296 |
| **Age** | less than 30 years old | 13.8 | 49.0 | 30.6 | 2.5 | 4.0 | 100 | 2386 |
|  | 30–39 years old | 12.4 | 50.9 | 30.4 | 2.6 | 3.8 | 100 | 2416 |
|  | 40–49 years old | 16.2 | 57.0 | 22.9 | 1.2 | 2.7 | 100 | 1527 |
|  | 50 years old above | 17.0 | 57.8 | 18.5 | 1.3 | 5.2 | 100 | 625 |
| ***Educational background*** | Junior high school diploma and below | 13.1 | 45.3 | 31.7 | 2.4 | 7.6 | 100 | 410 |
|  | High school diploma | 15.0 | 53.0 | 26.1 | 1.2 | 4.6 | 100 | 1581 |
|  | Junior college diploma | 14.3 | 51.3 | 28.7 | 2.4 | 3.4 | 100 | 3630 |
|  | Bachelor degree and above | 12.5 | 53.4 | 27.4 | 3.8 | 2.9 | 100 | 1322 |
| ***Sailing area*** | Ocean-going area | 15.3 | 52.5 | 27.1 | 2.1 | 2.9 | 100 | 4003 |
|  | Coastal area | 12.7 | 50.8 | 29.1 | 2.6 | 4.8 | 100 | 3068 |
| ***Types of ships working*** | Ordinary cargo ship | 14.2 | 52.2 | 27.9 | 2.0 | 3.6 | 100 | 4801 |
|  | Oil and Gas Ships | 13.4 | 50.9 | 28.1 | 3.4 | 4.1 | 100 | 2104 |
| ***Types of contract*** | Contract worker | 15.1 | 53.3 | 26.5 | 1.9 | 3.2 | 100 | 4073 |
|  | Dispatched workers | 14.6 | 50.8 | 27.7 | 3.2 | 3.6 | 100 | 1170 |
|  | Individual crew | 11.3 | 49.2 | 31.3 | 2.9 | 5.3 | 100 | 1828 |

**3-4 Participation in politics(%)**

| 3 Are you, or were you, a deputy to the National People's Congress at any levels? | | | | |  | 4 Have you ever been a member of the Chinese Political  Consultative Conference? | | | |
| --- | --- | --- | --- | --- | --- | --- | --- | --- | --- |
|  | |  | No | Yes |  | No | Yes | Total | N |
| Groups | | | 94.0 | 2.9 |  | 98.2 | 1.8 | 100 | 7296 |
| Age | less than 30 years old | | 91.5 | 4.3 |  | 97.2 | 2.8 | 100 | 2386 |
|  | 30–39 years old | | 94.4 | 2.8 |  | 98.3 | 1.7 | 100 | 2416 |
|  | 40–49 years old | | 96.6 | 1.9 |  | 99.4 | 0.6 | 100 | 1527 |
|  | 50 years old above | | 96.4 | 1.2 |  | 99 | 1.0 | 100 | 625 |
| ***Educational background*** | Junior high school diploma and below | | 92.8 | 4.0 |  | 98.9 | 1.1 | 100 | 410 |
|  | High school diploma | | 95.5 | 2.3 |  | 98.7 | 1.3 | 100 | 1581 |
|  | Junior college diploma | | 93.4 | 3.3 |  | 97.8 | 2.2 | 100 | 3630 |
|  | Bachelor degree and above | | 92.7 | 3.6 |  | 98.1 | 1.9 | 100 | 1322 |
| ***Sailing area*** | Ocean-going area | | 93.3 | 3.4 |  | 98 | 2.0 | 100 | 4003 |
|  | Coastal area | | 94.4 | 2.6 |  | 98.3 | 1.7 | 100 | 3068 |
| ***Types of ships working*** | Ordinary cargo ship | | 94.8 | 2.7 |  | 98.5 | 1.5 | 100 | 4801 |
|  | Oil and Gas Ships | | 91.4 | 3.9 |  | 97.2 | 2.8 | 100 | 2104 |
| ***Types of contract*** | Contract worker | | 95.1 | 2.5 |  | 98.7 | 1.3 | 100 | 4073 |
|  | Dispatched workers | | 90.1 | 4.7 |  | 96.6 | 3.4 | 100 | 1170 |
|  | Individual crew | | 93.2 | 3.2 |  | 97.7 | 2.3 | 100 | 1828 |

**5-6 Joining social organizations(%)**

|  | |  | A member of the labor union related to seafarers | A member of non-governmental organizations (NGO) related to navigation | N |
| --- | --- | --- | --- | --- | --- |
| Groups | | | 19.5 | 12.6 | 7296 |
| **Age** | less than 30 years old | | 14.6 | 17.2 | 2386 |
|  | 30–39 years old | | 15.8 | 9.6 | 2416 |
|  | 40–49 years old | | 28.4 | 9.4 | 1527 |
|  | 50 years old above | | 31.9 | 13.5 | 625 |
| ***Educational background*** | Junior high school diploma and below | | 11.3 | 20.1 | 410 |
|  | High school diploma | | 17.8 | 11.9 | 1581 |
|  | Junior college diploma | | 19.0 | 13.3 | 3630 |
|  | Bachelor degree and above | | 27.1 | 11.3 | 1322 |
| ***Sailing area*** | Ocean-going area | | 20.3 | 13.5 | 4003 |
|  | Coastal area | | 19.1 | 12.2 | 3068 |
| ***Types of ships working*** | Ordinary cargo ship | | 20.3 | 10.6 | 4801 |
|  | Oil and Gas Ships | | 18.5 | 18.1 | 2104 |
| ***Types of contract*** | Contract worker | | 28.1 | 11.3 | 4073 |
|  | Dispatched workers | | 12.2 | 15.9 | 1170 |
|  | Individual crew | | 6.0 | 14.5 | 1828 |

**7-12 The engagement in public affairs that Chinese merchant sailors participate in (%)**

|  |  | Have you ever done the following activities? | | | |  |
| --- | --- | --- | --- | --- | --- | --- |
|  |  | Never | Sometimes | Often | Total | N |
| 7 Have you given comments or suggestions to community leaders? | | 56.5 | 40.0 | 3.5 | 100 | 7296 |
| 8 Have you given advice or suggestions to the government? | | 87.5 | 10.6 | 1.9 | 100 | 7296 |
| 9 Have you made any comments through the media? | | 86.8 | 11.4 | 1.8 | 100 | 7296 |
| 10 Have you presented a petition? | | 84.9 | 13.0 | 2.1 | 100 | 7296 |
| 11 Have you made public comments on community issues? | | 66.6 | 31.2 | 2.2 | 100 | 7296 |
| 12 Have you made comments to people around you? | | 39.2 | 51.5 | 9.3 | 100 | 7296 |

**13 Merchant sailors’ response to obvious mistakes in shipping-related information or reports in the media(%)**

|  | | What would you do if you saw obviously wrong shipping related information or reports in the media?(multiple choice) | | | | | | |  |
| --- | --- | --- | --- | --- | --- | --- | --- | --- | --- |
|  | |  | Do nothing | Advice or suggestions to the unit leaders | Making suggestions to the media | Contact the media directly and point out the error | Clarify the mistake through social media such as QQ and Wechat | Other | N |
| Groups | | | 50.2 | 21.8 | 10.9 | 4.9 | 30.8 | 2.8 | 7296 |
| **Age** | less than 30 years old | | 45.5 | 24.4 | 14.3 | 6.2 | 34.3 | 2.9 | 2386 |
|  | 30–39 years old | | 51.6 | 19.0 | 9.2 | 4.2 | 31.8 | 3.5 | 2416 |
|  | 40–49 years old | | 53.5 | 19.6 | 9.1 | 4.1 | 29.0 | 3.0 | 1527 |
|  | 50 years old above | | 54.5 | 28.7 | 9.2 | 4.8 | 16.8 | 3.0 | 625 |
| ***Educational background*** | Junior high school diploma and below | | 41.2 | 34.7 | 11.4 | 7.1 | 25.3 | 2.3 | 410 |
|  | High school diploma | | 47.5 | 24.2 | 11.0 | 5.1 | 31.3 | 3.9 | 1581 |
|  | Junior college diploma | | 50.6 | 21.1 | 11.1 | 4.9 | 31.5 | 2.7 | 3630 |
|  | Bachelor degree and above | | 55.0 | 18.1 | 10.4 | 4.4 | 30.2 | 2.8 | 1322 |
| ***Sailing area*** | Ocean-going area | | 48.6 | 21.5 | 12.0 | 4.8 | 32.4 | 2.6 | 4003 |
|  | Coastal area | | 52.6 | 22.2 | 9.6 | 5.0 | 28.9 | 3.6 | 3068 |
| ***Types of ships working*** | Ordinary cargo ship | | 51.0 | 20.0 | 10.6 | 4.4 | 31.1 | 3.0 | 4801 |
|  | Oil and Gas Ships | | 49.1 | 25.7 | 11.7 | 6.4 | 30.6 | 3.0 | 2104 |
| ***Types of contract*** | Contract worker | | 52.4 | 52.4 | 19.8 | 10.3 | 4.6 | 30.7 | 4073 |
|  | Dispatched workers | | 45.3 | 45.3 | 27.5 | 13.2 | 6.6 | 31.6 | 1170 |
|  | Individual crew | | 48.9 | 48.9 | 23.4 | 11.2 | 4.9 | 30.5 | 1828 |

**14 Merchant sailors’ evaluation of the channel smoothness of social participation(%)**

|  |  | | Do you think the channels for participating in public affairs for sailors are smooth at present? | | | | | | |
| --- | --- | --- | --- | --- | --- | --- | --- | --- | --- |
|  |  | | Very smooth | Smooth | Not very smooth | Not smooth at all | Not sure | Total | N |
| Groups | | | 4.5 | 14.6 | 32.9 | 20.7 | 27.4 | 100 | 7296 |
| **Age** | | less than 30 years old | 5.7 | 16.9 | 34.0 | 19.0 | 24.3 | 100 | 2386 |
|  |  | 30–39 years old | 3.4 | 13.1 | 32.7 | 23.9 | 26.8 | 100 | 2416 |
|  |  | 40–49 years old | 4.2 | 13.2 | 33.6 | 19.4 | 29.5 | 100 | 1527 |
|  |  | 50 years old above | 5.1 | 14.6 | 27.3 | 17.0 | 36.0 | 100 | 625 |
| ***Educational background*** | | Junior high school diploma and below | 4.9 | 17.5 | 21.8 | 12.4 | 43.4 | 100 | 410 |
|  |  | High school diploma | 5.4 | 12.5 | 30.2 | 19.1 | 32.8 | 100 | 1581 |
|  |  | Junior college diploma | 4.3 | 15.6 | 34.6 | 20.3 | 25.2 | 100 | 3630 |
|  |  | Bachelor degree and above | 3.2 | 13.4 | 34.5 | 26.4 | 22.5 | 100 | 1322 |
| ***Sailing area*** | | Ocean-going area | 5.5 | 15.4 | 34.0 | 20.8 | 24.2 | 100 | 4003 |
|  |  | Coastal area | 2.9 | 13.2 | 31.0 | 20.9 | 31.9 | 100 | 3068 |
| ***Types of ships working*** | | Ordinary cargo ship | 4.5 | 14.0 | 33.1 | 21.2 | 27.2 | 100 | 4801 |
|  |  | Oil and Gas Ships | 4.4 | 15.4 | 32.0 | 20.2 | 28.1 | 100 | 2104 |
| ***Types of contract*** | | Contract worker | 4.7 | 14.2 | 33.0 | 20.8 | 27.4 | 100 | 4073 |
|  |  | Dispatched workers | 4.9 | 17.0 | 35.9 | 19.7 | 22.5 | 100 | 1170 |
|  |  | Individual crew | 3.5 | 13.4 | 30.2 | 21.5 | 31.4 | 100 | 1828 |

**15 Merchant sailors’ assessment of the influence of the labor union related to seafarers(%)**

|  |  | | What do you think of the influence of the labor union related to seafarers? | | | | | | |  |
| --- | --- | --- | --- | --- | --- | --- | --- | --- | --- | --- |
|  |  | | No influence | Weak influence | Influence | Great influence | Not sure | Total | N | |
| Groups | | | 26.4 | 51.3 | 9.1 | 2.6 | 10.6 | 100 | 7296 | |
| ***Age*** | | less than 30 years old | 23.2 | 50.1 | 11.3 | 3.0 | 12.5 | 100 | 2386 | |
|  |  | 30–39 years old | 29.3 | 50.6 | 8.1 | 2.0 | 10.1 | 100 | 2416 | |
|  |  | 40–49 years old | 26.6 | 55 | 7.2 | 2.8 | 8.5 | 100 | 1527 | |
|  |  | 50 years old above | 26.8 | 50.1 | 9.7 | 3.2 | 10.2 | 100 | 625 | |
| ***Educational background*** | | Junior high school diploma and below | 31.0 | 40.5 | 6.8 | 1.9 | 19.8 | 100 | 410 | |
|  |  | High school diploma | 26.0 | 50 | 8.7 | 2.9 | 12.3 | 100 | 1581 | |
|  |  | Junior college diploma | 26.1 | 51.9 | 9.9 | 2.5 | 9.6 | 100 | 3630 | |
|  |  | Bachelor degree and above | 27.1 | 53.7 | 8.1 | 2.8 | 8.4 | 100 | 1322 | |
| ***Sailing area*** | | Ocean-going area | 25.3 | 52.5 | 9.5 | 3.0 | 9.7 | 100 | 4003 | |
|  |  | Coastal area | 28.1 | 49.7 | 8.4 | 2.0 | 11.7 | 100 | 3068 | |
| ***Types of ships working*** | | Ordinary cargo ship | 25.6 | 52.4 | 9.2 | 2.7 | 10.1 | 100 | 4801 | |
|  |  | Oil and Gas Ships | 28.1 | 48.6 | 9.2 | 2.4 | 11.7 | 100 | 2104 | |
| ***Types of contract*** | | Contract worker | 24.8 | 54.4 | 10.1 | 3.2 | 7.6 | 100 | 4073 | |
|  |  | Dispatched workers | 28.9 | 47.2 | 9.6 | 2.2 | 12.1 | 100 | 1170 | |
|  |  | Individual crew | 28.5 | 47.7 | 6.4 | 1.5 | 15.9 | 100 | 1828 | |

**16 Merchant sailors’ assessment of the influence of the non-governmental organizations (NGO) related to navigation(%)**

|  |  | | What do you think of the influence of the non-governmental organizations (NGO) related to navigation? | | | | | |  |
| --- | --- | --- | --- | --- | --- | --- | --- | --- | --- |
|  |  | | No influence | Weak influence | Influence | Great influence | Not sure | Total | N |
| Groups | | | 23.6 | 50.2 | 11.0 | 2.5 | 12.7 | 100 | 7296 |
| **Age** | | less than 30 years old | 20.3 | 50.1 | 12.4 | 3.1 | 14.1 | 100 | 2386 |
|  |  | 30–39 years old | 27.3 | 50.4 | 8.8 | 1.7 | 11.9 | 100 | 2416 |
|  |  | 40–49 years old | 22.5 | 52 | 11.1 | 2.5 | 11.9 | 100 | 1527 |
|  |  | 50 years old above | 25.0 | 45.6 | 14.5 | 3.1 | 11.8 | 100 | 625 |
| ***Educational background*** | | Junior high school diploma and below | 28.4 | 41 | 11.1 | 2.5 | 17.0 | 100 | 410 |
|  |  | High school diploma | 22.7 | 48.7 | 11.3 | 2.8 | 14.5 | 100 | 1581 |
|  |  | Junior college diploma | 23.4 | 51 | 11.4 | 2.5 | 11.8 | 100 | 3630 |
|  |  | Bachelor degree and above | 24.3 | 52.3 | 9.6 | 2.1 | 11.8 | 100 | 1322 |
| ***Sailing area*** | | Ocean-going area | 22.5 | 51.3 | 11.7 | 2.6 | 11.8 | 100 | 4003 |
|  |  | Coastal area | 25.2 | 48.8 | 9.9 | 2.2 | 13.9 | 100 | 3068 |
| ***Types of ships working*** | | Ordinary cargo ship | 22.8 | 51.2 | 11.1 | 2.1 | 12.8 | 100 | 4801 |
|  |  | Oil and Gas Ships | 24.9 | 48.4 | 10.7 | 3.2 | 12.7 | 100 | 2104 |
| ***Types of contract*** | | Contract worker | 21.6 | 53 | 11.6 | 2.7 | 11.2 | 100 | 4073 |
|  |  | Dispatched workers | 25.4 | 48.4 | 10.6 | 2.7 | 12.9 | 100 | 1170 |
|  |  | Individual crew | 27.0 | 46.1 | 9.7 | 1.9 | 15.4 | 100 | 1828 |

**17 Merchant sailor’s understanding of the role of the labor union related to seafarers (%)**

|  |  | How well do you know about the labor union related to seafarers? | | | | |
| --- | --- | --- | --- | --- | --- | --- |
|  |  | Unknown | Know | Know very well | Total | N |
| Groups | | 83.1 | 15.6 | 1.3 | 100 | 7296 |
| Age | less than 30 years old | 86.2 | 12.2 | 1.6 | 100 | 2386 |
|  | 30–39 years old | 86.6 | 12.6 | 0.8 | 100 | 2416 |
|  | 40–49 years old | 77.3 | 21.4 | 1.3 | 100 | 1527 |
|  | 50 years old above | 71.4 | 26.5 | 2.1 | 100 | 625 |
| ***Educational background*** | Junior high school diploma and below | 89.7 | 8.4 | 1.8 | 100 | 410 |
|  | High school diploma | 82.5 | 16.3 | 1.2 | 100 | 1581 |
|  | Junior college diploma | 83 | 15.5 | 1.5 | 100 | 3630 |
|  | Bachelor degree and above | 81.8 | 17.5 | 0.7 | 100 | 1322 |
| ***Sailing area*** | Ocean-going area | 83.1 | 15.4 | 1.5 | 100 | 4003 |
|  | Coastal area | 83.1 | 15.9 | 1.0 | 100 | 3068 |
| ***Types of ships working*** | Ordinary cargo ship | 82.8 | 16.2 | 1.1 | 100 | 4801 |
|  | Oil and Gas Ships | 83.3 | 14.8 | 1.9 | 100 | 2104 |
| ***Types of contract*** | Contract worker | 79.2 | 19.4 | 1.4 | 100 | 4073 |
|  | Dispatched workers | 87.1 | 11.6 | 1.3 | 100 | 1170 |
|  | Individual crew | 88.8 | 10.5 | 0.7 | 100 | 1828 |

**18 Merchant sailor’s understanding of the role of the non-governmental organizations (NGO) related to navigation(%)**

|  | |  | How well do you know about the non-governmental organizations (NGO) related to navigation? | | | | |
| --- | --- | --- | --- | --- | --- | --- | --- |
|  | |  | Unknown | Know | Know very well | Total | N |
| Groups | | | 88.2 | 10.7 | 1.2 | 100 | 7296 |
| **Age** | less than 30 years old | | 89.5 | 8.9 | 1.6 | 100 | 2386 |
|  | 30–39 years old | | 91.3 | 8.2 | 0.5 | 100 | 2416 |
|  | 40–49 years old | | 84.5 | 14.5 | 1.0 | 100 | 1527 |
|  | 50 years old above | | 79.4 | 18.5 | 2.1 | 100 | 625 |
| ***Educational background*** | Junior high school diploma and below | | 87.1 | 10.8 | 2.1 | 100 | 410 |
|  | High school diploma | | 88.3 | 10.7 | 1.0 | 100 | 1581 |
|  | Junior college diploma | | 87.5 | 11.2 | 1.4 | 100 | 3630 |
|  | Bachelor degree and above | | 90.3 | 9.1 | 0.6 | 100 | 1322 |
| ***Sailing area*** | Ocean-going area | | 87.6 | 11.4 | 1.0 | 100 | 4003 |
|  | Coastal area | | 89.1 | 9.7 | 1.3 | 100 | 3068 |
| ***Types of ships working*** | Ordinary cargo ship | | 88.9 | 10.2 | 0.9 | 100 | 4801 |
|  | Oil and Gas Ships | | 86.2 | 12.0 | 1.8 | 100 | 2104 |
| ***Types of contract*** | Contract worker | | 87.6 | 11.2 | 1.2 | 100 | 4073 |
|  | Dispatched workers | | 88.1 | 10.6 | 1.3 | 100 | 1170 |
|  | Individual crew | | 89.3 | 9.8 | 0.9 | 100 | 1828 |

**19 Merchant sailors’ expectations of the services provided by social organizations（%）**

|  |  | | Which kinds of services provided by social organizations do you want? (multiple choice) | | | | | | | | | | |  |
| --- | --- | --- | --- | --- | --- | --- | --- | --- | --- | --- | --- | --- | --- | --- |
|  |  | | Providing information and technology | Policy advisory services | Providing employment opportunity | Providing advanced training | Research funding | Solving life difficulties | Reflecting problems to the government | Protection of rights | Providing opportunity to communicate with other social circles | Providing opportunity for maritime technology exchange | Other services | N |
| Groups | | | 49.8 | 34.0 | 48.6 | 26.7 | 10.7 | 28.9 | 19.1 | 49.9 | 26.1 | 25.0 | 2.0 | 7296 |
| **Age** | | less than 30 years old | 48.2 | 32.7 | 50.5 | 26.6 | 15.2 | 34.5 | 20.1 | 18.9 | 28.4 | 23.3 | 1.8 | 2386 |
|  |  | 30–39 years old | 47.3 | 35.6 | 50.0 | 27.5 | 10.0 | 27.8 | 19.7 | 50.3 | 28.1 | 25.8 | 2.2 | 2416 |
|  |  | 40–49 years old | 53.9 | 33.3 | 46.1 | 26.8 | 6.4 | 24.2 | 17.7 | 53.4 | 22.8 | 26.8 | 1.8 | 1527 |
|  |  | 50 years old above | 56.1 | 34.7 | 41.2 | 23.5 | 6.5 | 23.1 | 16.8 | 43.0 | 17.4 | 24.0 | 2.2 | 625 |
| ***Educational background*** | | Junior high school diploma and below | 45.6 | 32.3 | 56.5 | 26.5 | 6.7 | 31.8 | 13.9 | 41.6 | 19.4 | 18.3 | 2.3 | 410 |
|  |  | High school diploma | 47.8 | 32.0 | 51.9 | 26.0 | 8.6 | 30.7 | 16.2 | 50.0 | 22.4 | 22.2 | 2.4 | 1581 |
|  |  | Junior college diploma | 49.9 | 35.0 | 49.8 | 28.3 | 12.2 | 29.7 | 20.8 | 50.8 | 27.8 | 26.4 | 1.8 | 3630 |
|  |  | Bachelor degree and above | 53.6 | 36.8 | 39.5 | 25.0 | 11.5 | 24.6 | 20.1 | 49.3 | 28.8 | 26.7 | 2.6 | 1322 |
| ***Sailing area*** | | Ocean-going area | 50.1 | 34.3 | 44.5 | 27.4 | 12.3 | 28.5 | 19.2 | 49.8 | 28.5 | 25.4 | 1.9 | 4003 |
|  |  | Coastal area | 49.6 | 34.3 | 54.6 | 26.3 | 8.8 | 29.7 | 19.2 | 50.1 | 23.3 | 24.4 | 2.3 | 3068 |
| ***Types of ships working*** | | Ordinary cargo ship | 51.1 | 33.6 | 47.7 | 26.8 | 11.2 | 29.0 | 19.5 | 49.8 | 26.8 | 24.8 | 2.1 | 4801 |
|  |  | Oil and Gas Ships | 47.2 | 36.5 | 51.5 | 26.9 | 10.5 | 28.7 | 18.6 | 50.2 | 25.7 | 25.7 | 2.1 | 2104 |
| ***Types of contract*** | | Contract worker | 52.7 | 34.5 | 43.7 | 27.3 | 11.2 | 28.3 | 19.0 | 49.9 | 27.5 | 26.7 | 1.9 | 4073 |
|  |  | Dispatched workers | 46.1 | 35.4 | 47.8 | 26.2 | 13.1 | 28.6 | 17.3 | 47.6 | 28.3 | 22.5 | 2.4 | 1170 |
|  |  | Individual crew | 45.4 | 33.4 | 60.4 | 26.8 | 8.5 | 30.7 | 20.5 | 50.9 | 21.8 | 23.0 | 2.2 | 1828 |
