# Supplementary material for: Willingness to engage in and current status of social participation among Chinese merchant sailors
Source: PLoS One. 2020 Nov 25;15(11):e0242888. doi: 10.1371/journal.pone.0242888 (PMC7688133; doi:10.1371/journal.pone.0242888)
Supplement: S2 File — (DOCX) [file pone.0242888.s002.docx]

**Questionnaire in study**

**中国海员社会参与现状调查问卷（Chinese version）**

亲爱的朋友，您好：

谢谢您在百忙之中协助调查研究。本调查旨在全面、客观地了解目前我国船舶技术人员群体的社会参与状况和需求，反映船舶技术人员的意见、呼声，维护广大船舶技术人员的合法权益，为党和政府及相关部门制定有关政策提供参考。

问卷为不记名填写，我们承诺对您的个人信息予以**严格保密**，请按照您的实际情况和真实想法填答本问卷，答案没有“对”、“错”之分。

十分感谢您的支持与合作！

1. **个人基本情况**

A1．您的性别是： 1男 2女

A2．您的出生年月： 年 月

A3．从事海员工作前，您来自： 1城市 2乡镇 3农村

A4．您的最高学历是：

1本科及以上 2大专/高职 3 高中/中专/技校 4初中及以下

A5. 您的工龄是：

1 5年及以下 2 6-10年 3 11-20年 4 21年及以上

A6．近三年，您主要工作的船舶类型是：

1 客滚轮 3油化或液化气船

A7. 您的劳务合同属于：

1 劳动合同工 2 劳务派遣工 3 个体船员

A8. 近三年，您主要工作的航区：

1 远洋航区 2近海航区

**二、社会参与情况**

B1您愿意参与单位的公共事务管理吗？

1 非常愿意　　2 比较愿意　　3 不太愿意　　4 完全不愿意　　5 不知道

B2 对于近年来国家出台的政策方针，您是否关注？

1 非常关注　　2 比较关注　　3 不太关注　　4 完全不关注　　5 不知道

B3 您当选过各级人大代表吗？

1 有 2 没有

B4 您当选过各级政协委员吗？

1 有 2 没有

B5 您是航海相关的工会会员吗？

1是 2 不是

B6 您是航海相关的非政府组织成员吗？

1是 2 不是

**您是否做过以下活动？在合适的数字上划“Ο”。**

| 项目 | **没有**  **0** | **有时**  **1** | **经常**  **2** |
| --- | --- | --- | --- |
| B7 向单位领导（部门）提建议/意见 | **0** | **1** | **2** |
| B8 向政府提建议/意见 | **0** | **1** | **2** |
| B9 向新闻媒体提建议/意见 | **0** | **1** | **2** |
| B10 参加上访/请愿 | **0** | **1** | **2** |
| B11 就单位的管理问题公开发表意见 | **0** | **1** | **2** |
| B12 向周围人或在朋友圈发表意见 | **0** | **1** | **2** |

B13 如果在媒体上看到了您认为明显错误的与航运有关的信息或报道，您一般会怎么办？【可多选】

1 不予理睬　　2 向相关管理部分反映　　3 与该媒体联系，指出错误

4 通过媒体向公众澄清错误　　5 运用QQ或微信向朋友圈澄清错误

6 其他

B14 您觉得目前参政议政或参与公共事务的渠道通畅吗？

1 非常通畅　　2 比较通畅　　3 不太通畅　　4 很缺乏　　5 不清楚

B15 您认为航海相关的工会所发挥的影响力如何？

1非常有影响 2 有影响 3 影响较弱 4 没有影响 5 不清楚

B16 您认为航海相关的非政府组织所发挥的影响力如何？

1非常有影响 2 比较有影响 3 影响较弱 4 没有影响 5 不清楚

B17 您对航海相关的工会了解程度如何？

1 非常了解 2 比较了解 3 不了解

B18 您对航海相关的非政府组织了解程度如何？

1 非常了解 2 比较了解 3 不了解

B19您希望从这些社会组织团体中获得哪些帮助？【可多选】

1 保障权益 2 资助研究 3 向政府反映意见 4 政策咨询服务 5 解决生活困难 6 进修培训服务 7 提供与社会各界交流的机会 8 提供航海技术培训机会 9信息、技术服务 10就业服务

11 其他服务

**Questionnaire on Chinese seafarers' social participation**

**(English version)**

**【Instructions】**

Dear friends,

Thank you for your help in the investigation. The purpose of this survey is to understand the social participation status and needs of Chinese seafarers, reflect the opinions and voices of seafarers, safeguard the legitimate rights and interests of the majority of seafarers, and provide reference for the government and relevant departments to formulate relevant policies.

The questionnaire is to be filled in anonymously. We promise to keep your personal information confidential. Please fill in and answer this questionnaire according to your actual situation and real ideas. There is no "right" or "wrong" answer.

Thank you very much for your support and cooperation!

**【Basic individual information】**

1. Your gender is:

1=male

2=female

1. Your birthday is: Month Year
2. Your residence

1=city

2= small urban area

3= rural area

1. Your highest education is:

1=bachelor degree and above

2= junior college diploma

3=high school diploma

4= junior high school diploma and below

1. Your length of career is:

1=5 years or less

2= 6–10 years

3= 11–20 years

4= 21 years or more

1. The types of ships you work in is:

1=ordinary cargo ship

2= oil and Gas Ships

1. The types of your contract is:

1= contract worker

2= dispatched workers

3= individual crew

1. In the past three years, your main work areas is:

1= ocean-going area

2=coastal area

**【Detailed items】**

1. Would you like to participate in public affairs in your community?

1 = very willing

2 = willing

3 = unwilling

4 = completely unwilling

5 = not sure

(2) Are you concerned about the national policies introduced in recent years?

1= great concern

2= concern

3= little concern

4= no concern

5= not sure

(3) Are you, or were you, a deputy to the National People's Congress at any levels?

1= yes, 2= no

(4) Have you ever been a member of the Chinese Political Consultative Conference?

1= yes, 2= no

(5) Are you a member of the labor union related to Chinese seafarers?

1= yes, 2= no

(6) Are you a member of non-governmental organizations (NGO) related to navigation in China?

1= yes, 2= no

(7-12) Have you ever done the following activities?

| 项目 | Never  **0** | Sometimes  **1** | Often  **2** |
| --- | --- | --- | --- |
| 7 Have you given comments or suggestions to community leaders? | **0** | **1** | **2** |
| 8 Have you given advice or suggestions to the government? | **0** | **1** | **2** |
| 9 Have you made any comments through the media? | **0** | **1** | **2** |
| 10 Have you presented a petition? | **0** | **1** | **2** |
| 11 Have you made public comments on community issues? | **0** | **1** | **2** |
| 12 Have you made comments to people around you? | **0** | **1** | **2** |

(13) What would you do if you saw obviously wrong shipping related information or reports in the media?(multiple choice)

1 = do nothing,

2= advice or suggestions to the unit leaders,

3= making suggestions to the media,

4= contact the media directly and point out the error

5= clarify the mistake through social media such as QQ and Wechat

6= other

(14) Do you think the channels for participating in public affairs for sailors are smooth at present?

1 = very smooth

2 = smooth

3 = not very smooth

4 = not smooth at all

5= not sure

1. What do you think of the influence of the labor union related to seafarers?

1= great influence

2= influence

3= weak influence

4=no influence

5=not sure

1. What do you think of the influence of the non-governmental organizations (NGO) related to navigation?

1= great influence

2= influence

3= weak influence

4=no influence

5=not sure

1. How well do you know about the labor union related to seafarers?

1= know very well

2= know

3=unknown

1. How well do you know about the non-governmental organizations (NGO) related to navigation?

1= know very well

2= know

3=unknown

1. Which kinds of services provided by social organizations do you want? (multiple choice)

1= protection of rights

2= research funding

3= reflecting problems to the government

4= policy advisory services

5= solving life difficulties

6=providing advanced training

7= providing opportunity to communicate with other social circles

8= providing opportunity for maritime technology exchange

9= providing information and technology

10= providing employment opportunity

11= other services
